# Ultra-hypofractionation for node-positive prostate cancer: pushing boundaries and redefining standards

**DOI:** 10.1038/s41391-025-00976-7

**Published:** 2025-05-08

**Authors:** Jennifer Le Guévelou, Mathilde Jeandin, Antonio Angrisani, Davide Giovanni Bosetti, Mohamed Shelan, Thomas Zilli

**Affiliations:** 1 Laboratoire de traitement du signal et de l’image, Rennes University, Rennes, France; 2 Department of Geriatry, Geneva University Hospital, Geneva, Switzerland; 3 Department of Radiation Oncology, Oncology Institute of Southern Switzerland, EOC, Bellinzona, Switzerland; 4 Department of Radiation Oncology, Inselspital, Bern University Hospital, University of Bern, Bern, Switzerland; 5 Faculty of Biomedical Sciences, Università della Svizzera italiana, Lugano, Switzerland; 6 Faculty of Medicine, University of Geneva, Geneva, Switzerland

**Keywords:** Cancer therapy, Outcomes research

## Abstract

Radiotherapy is a cornerstone in the management of node-positive prostate cancer. Advances in imaging modalities and radiation therapy techniques have led to the evolution of treatment standards for this patient population. This review aims to explore the therapeutic advancements of the past decade, with a focus on the role of ultra-hypofractionated radiotherapy in node-positive prostate cancer.

Management of prostate cancer with positive loco-regional nodal disease has always represented a challenging clinical situation, with lifelong androgen deprivation therapy (ADT) long being proposed as a standard but palliative approach [1]. In the 2006–2011 period, the use of a local therapy with either radical prostatectomy (RP) or radiotherapy (RT) was proposed to only 40% of patients, as reported by the National Cancer Database registry (NCDB) [2]. Over the past decade, a paradigm shift has occurred, with locoregional RT increasingly recognized as a curative treatment option when combined with ADT. In a subgroup of patients with node-positive prostate cancer within the control arm of the STAMPEDE trial, the addition of RT to long-term ( ≥ 2 years) ADT has been shown to improve failure free-survival (FFS), with 2-year FFS reaching 81% and 53%, respectively [3]. Concomitantly, the integration of androgen receptor pathway inhibitors (ARPIs) alongside RT has become the new standard of care for managing patients with very high-risk prostate cancer and those with node-positive disease on conventional imaging. This approach has demonstrated to improve clinical outcome compared to long-term ADT alone, with a 6-year OS reaching 86% by adding two years of abiraterone acetate compared to a 77% rate with 36 months of ADT (*p* < 0.001) [4]. Based on the recent international guidelines, long-term ADT with 2 years of abiraterone acetate plus prednisone and RT including irradiation of the nodal pelvic regions up to 45–54 Gy in equivalent dose in 2 Gy per fraction should be considered one of the treatment option for this patient population [5–7].

Integration of new imaging techniques like prostate specific membrane antigen (PSMA) computed tomography (PET/CT) in the diagnostic workflow has certainly enlarged the number of patients diagnosed with a node positive disease. Thanks to the higher accuracy of PSMA PET/CT compared to conventional imaging for the evaluation of nodal disease [8], approximately 20% of patients with high-risk localized disease are expected to be classified as node positive in the PSMA era. While the role of RT with long-term ADT remains one of the cornerstone treatments, the benefit of adding ARPIs, well established for clinically node positive disease based on conventional imaging (short axis dimension of the node superior to 8–10 mm) [3], remains less clear in case of sub-centimetric but PSMA positive nodes. The PATRON study (NCT04557501), randomizing men between PET/CT-guided RT planning (with treatment-intensification on newly identified disease) and conventional imaging-guided RT will provide insightful data regarding the impact of intensified RT guided by PSMA PET/CT.

Concerning treatment optimization, several strategies have been developed in the past decade in order to increase the therapeutic ratio in patients with node-positive prostate cancer treated with curative RT (Fig. 1). The impact of dose escalation to the involved pelvic nodes has been investigated by some authors. Tsuchida et al. retrospectively reported a benefit in terms of metastasis-free survival (MFS) with dose-escalation over 60 Gy, compared with standard-dose whole pelvis RT (WPRT) (7-year MFS: 90.6% vs 62.8%, *p* = 0.023) [9]. Fonteyne et al. showed a promising 3-year biochemical relapse-free survival (bRFS) in patients treated with WPRT and a simultaneous integrated boost (SIB) delivered to the gross pelvic lymph nodes up to 65 Gy in 25 fractions, reaching 63% [10]. Despite these data, no prospective randomized control trial has been led to assess the oncological and toxicity outcomes with the addition of dose escalation on positive lymph nodes. Therefore, dose escalation to clinically positive nodes should only be considered if it is deemed to be isotoxic [6, 7]. As dose-escalation on positive lymph nodes has been suggested to positively impact MFS, to date this strategy has not been tested in a population of patients receiving ARPI. In the STAMPEDE trial, a large proportion of patients receiving abiraterone acetate for node-positive prostate cancer did not receive pelvic radiotherapy, and none received dose-escalation on positive nodes (staged with conventional imaging). Whether or not there is a benefit to add ARPI in patients receiving WPRT and dose-escalation on positive lymph nodes is unknown. Ongoing trials like the ALADDIN (GETUG-P17), testing the addition of Darolutamide to long-term ADT and dose-escalated RT in patients with node-positive prostate cancer staged with PSMA PET/CT will certainly help to provide additional evidence on the role of ARPIs in this setting.

**Fig. 1:**
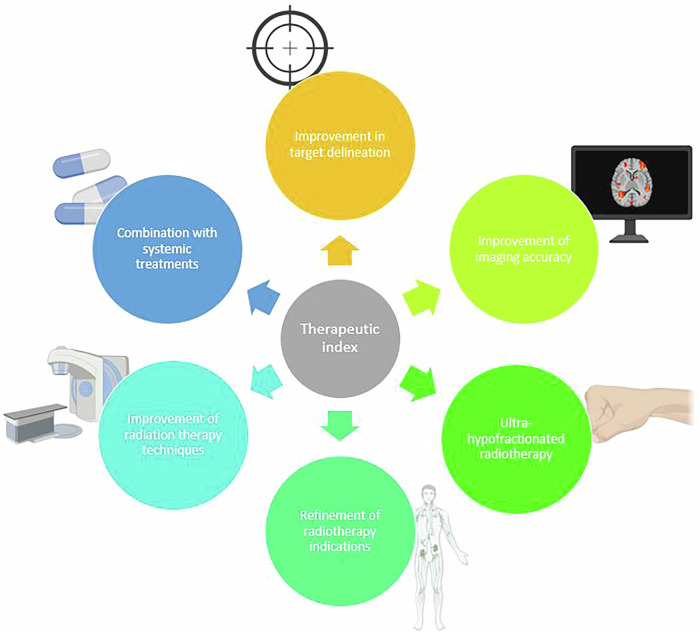
Strategies enabling an improvement of the therapeutic index for the management of node-positive prostate cancer.

In analogy with localized disease where stereotactic body radiotherapy (SBRT) is a standard, a 5-fraction ultra-hypofractionated schedule has been recently explored also in patients with node-positive disease [11–15] (Table 1). Most of these trials implemented dose-escalation on positive lymph nodes [12–15]. Murthy et al. recently reported the results of a prospective database with ultra-hypofractionated RT performed in patients with either high-risk, very high-risk, and node positive prostate cancer [12]. Overall, biochemical progression -free survival (bPFS) reached 94% at 18 months. Late grade 2 genitourinary (GU) and gastrointestinal (GI) toxicity was reported in 4.5% and 4% of the patients, respectively, suggesting the safety of ultra-hypofractionation including WPRT. Late grade 3 GU toxicity was observed in 2.5% of the patients, and consisted in urethral strictures, probably due to the relatively high proportion of patients with a previous history of transurethral resection of the prostate (TURP) (17%). Telkhade et al. also demonstrated excellent toxicity outcomes in a prospective database including 60 patients with node-positive prostate cancer [15]. At a median follow-up of 30 months, grade≥2 GU and GI toxicity was observed in only 6.6% and 8.3% of the patients, respectively. The prospective, randomized, non-inferiority phase III PRIME trial recently randomized patients with high-risk, very high-risk, or node-positive prostate cancer (22.9% of participants) to receive either a moderate hypofractionated RT schedule or a ultra-hypofractionated RT to the prostate and the whole pelvis. Acute toxicity outcomes presented at ESTRO 2023 showed a favorable toxicity profile, with no difference observed in severe GU or GI toxicity. Quality of life for urinary and bowel domains as assessed by EORTC QLQ C-30 an PR-25 remained overall stable and comparable between the two arms. Long-term toxicity and outcome results are expected in the next years. All the studies proposing ultra-hypofractionation for node-positive prostate cancer performed the treatment every-other-day, as suggested within the ASTRO/ASCO/AUA evidence-based guideline [16].

**Table 1:** Studies assessing the outcomes after ultra-hypofractionated prostate and whole pelvic radiotherapy (WPRT) for node-positive prostate cancer.

Study	Design of the study	Number of patients	RT technique	RT dose	PTV margins	ADT	Median follow-up	Acute toxicity outcomes	Late grade ≥ 2 toxicity outcomes	Oncological outcomes
Murthy et al. [12]	Prospective database	68 pts (30%HR, 16% vHR, 54% cN1)	Tomotherapy or VMAT with fiducials	Prostate: 35–37.5 Gy/5fx WPRT: 25 Gy/5fx cN1: 35- 37.5 Gy/5fx (SIB^a^) Every other day	CTV + 5 mm except posteriorly (3 mm)	cN0: 24 months cN1: lifelong	18 months	G2 GU: 12% G3 GU: 1.5% G2 GI: 3%	G2 GU : 4.5% G3 GU: 2.5% G2 GI: 4%	18-months bPFS: 94%
Pinitpatcharalert et al., 2018	Retrospective	23 pts (87%HR, 13% cN1)	VMAT with fiducials ± hydrogel rectal spacer	Prostate: 37.5–40 Gy/5fx WPRT: 25 Gy/5fx Every other day	CTV + 5 mm except posteriorly (3 mm) WPRT: CTV + 5–8 mm	18 months	19 months	G2 GU: 36.4% G3 GU toxicity: 4.5% G2 + GI: 0%	G2 GU: 27.3% G3 GU: 4.5% G2 GI: 9.1%	2-year bPFS: 95.7%
Telkhade et al. [15]	Prospective database	60 pts (100% cN1)	VMAT	Prostate: 35–37.5 Gy/5fx WPRT: 25 Gy /5fx cN1: 35 Gy /5fx (SIB^a^) Every other day	CTV + 5 mm	30% orchidectomy 70% long-term ADT	30 months	G2 GU: 8.3% G3 + GU: 3.3% G2 GI: 11.7%	G2 GU: 3.3% G3 GU: 3.3% G2 GI: 8.3%	3-year bPFS: 77%
Kishan et al. [14]	Prospective phase III trial	156 pts (18% FIR, 42% UIR, 23% HR, 10% vHR, 7% cN1)	MRgRT vs CT-based VMAT	Prostate: 40 Gy/5fx ± DIL boost 42 Gy/5fx WPRT: 25 Gy/5fx cN1: 35 Gy/5fx (SIB^a^) Every other day	CTV + 4 mm for CT-guided CTV + 2 mm for MRgRT	68% of the patients based on NCCN	24 months	G2 + GU: MRgRT 24.4% vs CT-guided 43.4% G2 + GI: MRgRT 0%, vs CT-guided 10.5%	G2 + GU: MRgRT 27% vs CT-guided 51% G2 + GI: MRgRT 1.4% vs CT-guided 9.5%	NR
Murthy et al., ESTRO 2023	Prospective phase III trial	307 pts (21% cN1)	Tomotherapy or VMAT	Moderate hypofractionation Prostate: 62–68 Gy/20–25fx, WPRT: 44–50 Gy/20–25fx cN1: 60–66 Gy/20–25fx (SIB^a^) Ultra-hyprofractionated Prostate: 36.25 Gy/5fx WPRT: 25 Gy/5fx cN1: up to 35 Gy/5fx (SIB^a^) Every other day	CTV + 5 mm	At least 24 months	90 days	Moderate hypofractionation G2 + GU: 34.5% G2 + GI: 17.9% Ultra-hypofractionation G2 + GU: 32.5% G2 + GI: 16.9%	NR	NR

*WPRT* whole pelvic radiotherapy, *fx* fractions, *LINAC* linear accelerator, *pts* patients, *HR* high-risk, *UIR* unfavorable intermediate risk, *DIL* dominant intraprostatic lesion, *GU* genitourinary, *GI* gastrointestinal, *bPFS* biochemical progression-free survival, *VMAT* volumetric arc therapy, *vHR* very high-risk, *FIR* favorable intermediate-risk, *MRgRT* magnetic resonance guided radiotherapy, *NR* not reported, *MRgRT* magnetic resonance guided radiotherapy, *CTV* clinical target volume, *cN1* clinically node positive, *CT* computed tomography, *G2 +*  grade superior or equal to 2, *SIB* simultaneous integrated boost

^a^Simultaneous integrated boost (SIB) involves the irradiation of all target volumes during the entire treatment period, different dose levels being delivered simultaneously in different target volumes.

The optimal dose to be delivered to both whole pelvis and positive lymph nodes is to date undetermined, for both normofractionated, moderately hypofractionated and ultra-hypofractionated schedules, The efficacy of a 25 Gy/5fx schedule for prophylactic pelvic nodal irradiation has been recently assessed within a pooled analysis performed by the SHARP consortium, reporting an overall pelvic control rate reaching 98.2% in men with high-risk prostate cancer at a median follow-up of 51 months [17]. Regarding toxicity, a recent meta-analysis reported a good tolerability of ultra-hypofractionation for treating both the prostate (median dose 40 Gy/5fx) and pelvic lymph nodes (median dose 25 Gy/5fx) in men with high-risk prostate cancer, reporting at a median follow-up of 3 years late grade ≥2 GU and GI toxicity rates of 29% and 13%, respectively [18]. While data from the PRIME trial do not suggest an increased risk of toxicity with ultra-hypofractionated RT compared with moderately fractionated RT, these results are consistent with early results from the HOPE trial comparing brachytherapy boost with either ultra-hypofractionated vs normofractionated WPRT [19]. Toxicity outcomes from studies performing ultra-hypofractionated radiotherapy for node-positive prostate cancer with or without dose-escalation on macroscopic lymph nodes (Table 1) compare favorably with these results. As limited data are available with 5-fraction on ultra-hypofractionated schedules including WPRT, the widespread adoption of this schedule is still subjected to prospective evaluation [16]. The FASTR trial published in 2015, which enrolled a vulnerable and elderly population treated with 40 Gy to the prostate and 25 Gy to the pelvic lymph nodes in a 5-fraction weekly schedule, was prematurely terminated due to unacceptable levels of late GU and GI toxicity [20]. Rectal bleeding was associated with the volume of rectum receiving high doses of radiation due to inclusion of a large part of seminal vesicles, a CT-based only planning, and no use of fiducials for repositioning. The SATURN phase I-II trial, including adjusted RT doses, stringent dose constraints, fiducial tracking, reduced PTV margins, and refined patient selection [21] showed a rate of late grade 2 GU and GI of 52% and 32%, respectively. No grade ≥3 toxicities were reported. Interestingly, a post-hoc analysis comparing patients treated in the SATURN and pHART8 trials revealed that adding WPRT significantly improved biochemical control and 4-year prostate-specific antigen (PSA) response rates without increasing late GU or GI toxicity [22]. To date, none of the modern randomized controlled trials testing the addition of WPRT in men with prostate cancer demonstrated WPRT to be associated with an increased risk of severe toxicity [23, 24].

As toxicity associated with prostate and pelvic SBRT is predominantly driven by prostate irradiation, further attempts to mitigate the dose delivered to the organs at risk are needed when delivering ultra-hypofractionation. Use of devices like fiducial-based tracking systems, recto-prostatic spacers or endorectal balloons have been proposed as possible strategies to reduce the potential toxicity of ultra-hypofractionation. Reduction of treatment margins possible with magnetic resonance-guided radiotherapy (MRgRT) or adaptive RT solutions has demonstrated a significant reduction in the risk to develop acute and late toxicity compared with standard CT-based RT using larger PTV margins. The phase III MIRAGE trial, which included patients with high-risk, very high-risk, or node-positive prostate cancer, demonstrated significantly lower cumulative 2-year incidence rates of late grade ≥2 toxicity with MRgRT and margin reduction compared to standard CT-based radiotherapy. GU toxicity rates were 27% versus 51% (*p* = 0.004), and GI toxicity rates were 1.4% vs. 9.5% (*p* = 0.025), both favoring MRgRT [13].

The benefit of elective pelvic RT compared with metastases-directed therapy has recently been highlighted by the STORM PEACE-V trial, conducted in a population of patients with pelvic nodal metachronous oligorecurrence, reporting a 3-year bRFS respectively reaching 69% and 47% [25]. In both metachronous de novo and oligorecurrent node-positive prostate cancer, standardization of pelvic target volumes is critical. While the most recent consensus on target volumes for elective pelvic irradiation agreed to include common iliac stations [26, 27], resulting in an expected coverage of 93% of nodal recurrences [28], patients with de novo prostate diagnosed with common iliac nodal involvement are still classified as having metastatic nodal disease (M1a). Yet, oncological outcomes of these patients are similar to those with N1 disease when treated with WPRT and nodal boost [29]. In a prospective database of patients with N1 and M1a disease mostly staged with PSMA PET/CT (75%) the 5-year bRFS was similar between the groups (N1: 77.4% vs. M1a: 70.4%; *p* = 0.43), as were the 5-year MFS (N1: 86.9% vs. M1a: 79.4%; *p* = 0.23), and OS (N1: 92.6% vs. M1a: 90.1%; *p* = 0.80). These results underscore the potential benefit of a local treatment approach even for patients harboring oligometastatic nodal disease. Whether these conclusions can be extended to patients with para-aortic lymph node involvement is currently being investigated in the PEARLS trial (ISRCTN36344989). This trial randomizes extended field RT to the para-aortic nodal stations, with moderate hypofractionation, in patients with either pelvic or para-aortic node-positive prostate cancer, and will also evaluate the role of dose-escalated radiotherapy performed to involved nodes in patients with para-aortic nodal metastases.

In conclusion, despite the stage migration driven by advanced imaging techniques, local curative approaches remain a cornerstone in managing node-positive prostate cancer. Looking ahead, strategies involving ultra-hypofractionated pelvic irradiation have shown promising outcomes in the treatment of these patients. While dose escalation is now feasible due to state-of-the-art technologies, de novo node-positive prostate cancer continues to represent a disease status with poor prognosis, underscoring the critical need for further treatment intensification strategies.
